# Analysis of Clinical Characteristics and Polysomnography Indicators of Obstructive Sleep Apnea–Hypopnea Syndrome Patients Based on Sleep Perception Types

**DOI:** 10.3389/fneur.2020.00988

**Published:** 2020-09-11

**Authors:** Yishu Liu, Huiwen Tan, Yue Yu, Yin Zeng, Li Xiao

**Affiliations:** ^1^Department of Pulmonary and Critical Care Medicine, Shengjing Hospital of China Medical University, Shenyang, China; ^2^The Sleep Medicine Center, Shengjing Hospital of China Medical University, Shenyang, China

**Keywords:** OSAHS, sleep perception, polysomnography, breathing disease, clinic characteristics

## Abstract

**Purpose:** The aim of this study was to explore the clinical characteristics of different sleep perception types of obstructive sleep apnea–hypopnea syndrome (OSAHS) patients and to analyze the correlation between sleep perception and polysomnography (PSG) indicators in OSAHS patients.

**Methods:** We retrospectively analyzed 355 patients diagnosed with OSAHS by PSG at the Sleep Medicine Center of Shengjing Hospital of China Medical University. Patients saw a doctor due to snoring and daytime sleepiness from March 2017 to March 2018. We excluded the patients who are <18 years old, had a history of OSAHS treatment, had other sleep and psychiatric disorders, and could not provide complete data. According to the patients' explanation, medical history, PSG indicators, and morning questionnaire after PSG, the patients were divided into normal sleep perception (NSP), positive sleep perception abnormality (PSPA), and negative sleep perception abnormality (NSPA). We analyze the demographic characteristics and PSG of the three groups with ANOVA and non-parametric tests. In addition, we conducted correlation analysis between sleep perception and PSG indicators.

**Results:** Of OSAHS patient, 55.5% had sleep perception abnormalities, of which 35.5% were positive-perception abnormalities and 20% were negative-perception abnormalities. From the analysis of PSG indicators, the sleep perception abnormality was related to the frequency of spontaneous arousal of the patient (*P* = 0.003) and was not related to the slight arousal caused by respiratory events, oxygen desaturations, and limb movement events. OSAHS patients with PSPA had a higher oxygen desaturation index (*P* = 0.046) but no significant difference in *post hoc* test. PSPA group had significantly lower rapid eye movement (REM) latency and sleep efficiency and more wake after sleep onset (WASO) than had the other sleep perception groups. Multivariate linear regression analyses after adjusting for age and sex revealed that sleep perception was related to lowest oxygen saturation (LSaO_2_), TS90%, sleep efficiency, and WASO.

**Conclusion:** Sleep perception abnormality is common in OSAHS patients. OSAHS patients with different sleep perception types have different PSG profiles. The OSAHS patients with PSPA have more severe hypoxia levels at night that require timely personalized treatment.

## Introduction

Obstructive sleep apnea–hypopnea syndrome (OSAHS) is a common sleep-related breathing disease. The pathophysiology is characterized by intermittent hypoxia and sleep fragmentation at night (1). It has become a hot topic due to its close link to cardiovascular, cerebrovascular, and metabolic diseases; other multiple organ system damage; and even sudden death. From the patients' medical history, we found that although they slept all night, the patients still felt fatigued and sleepy during the daytime. This phenomenon prompts us to pay more attention to the sleep perception of the patients with OSAHS.

Sleep perception refers to the self-assessment of an individual's sleep state and mainly includes a subjective description of the quantity and quality of his or her sleep. This subjective evaluation has become one of the foundations for the diagnosis and evaluation of sleep disorders (2). The overall sleep quality depends on whether the sleep time can meet the physical and mental demands of the body during daytime. Therefore, subjective perception and judgment of sleep time are the basis for sleep perception, including sleep latency (SL) and total sleep time (TST). Most studies toward sleep perception focus on insomnia because sleep misperception makes patients with insomnia believe that they are getting insufficient sleep, which subsequently causes their condition to aggravate. Studies suggest that OSAHS patients, in contrast to insomnia patients, have intact sleep perception, even if they sleep less (3). However, it was found that there were significant differences between subjective and objective sleep parameters through analysis of subjective assessment of sleep parameters in patients with OSAHS and objective sleep parameters detected by polysomnography (PSG) (4). Abnormal sleep perception mainly refers to the discrepancy between subjective assessment and objective detection, which can be divided into positive discrepancy and negative discrepancy. Patients with insomnia often show negative discrepancy, which is overestimation of SL, underestimation of TST, and sleep efficiency. Otherwise, overestimation of TST is positive discrepancy (5). Research has provided relatively little insight into different sleep perception types in OSAHS patients, especially positive discrepancy.

The aim of this study was to investigate the clinical characteristics, PSG indicators, and sleep structure characteristics of OSAHS patients with different sleep perception types. We also discussed the related analysis of sleep perception and PSG indicators in order to provide more reliable evidence for sleep assessment and clinical classification of patients with OSAHS.

## Methods

### General Information

The study subjects were patients who were treated at the Sleep Medicine Center of Shengjing Hospital of China Medical University from March 2017 to March 2018. Their main symptoms were snoring and daytime sleepiness.

Exclusion criteria for patients was as follows: (1) younger than 18 years; (2) had a history of OSAHS treatment; (3) had any sleep disorders such as restless legs syndrome (RLS), narcolepsy, and parasomnia; (4) had serious psychiatric disorders; and (5) could not provide complete data. Altogether, 355 patients were diagnosed with OSAHS by PSG, 284 males and 71 females, with an average age of 43.23 ± 12.84 years.

### Methods

**Figure 1:**
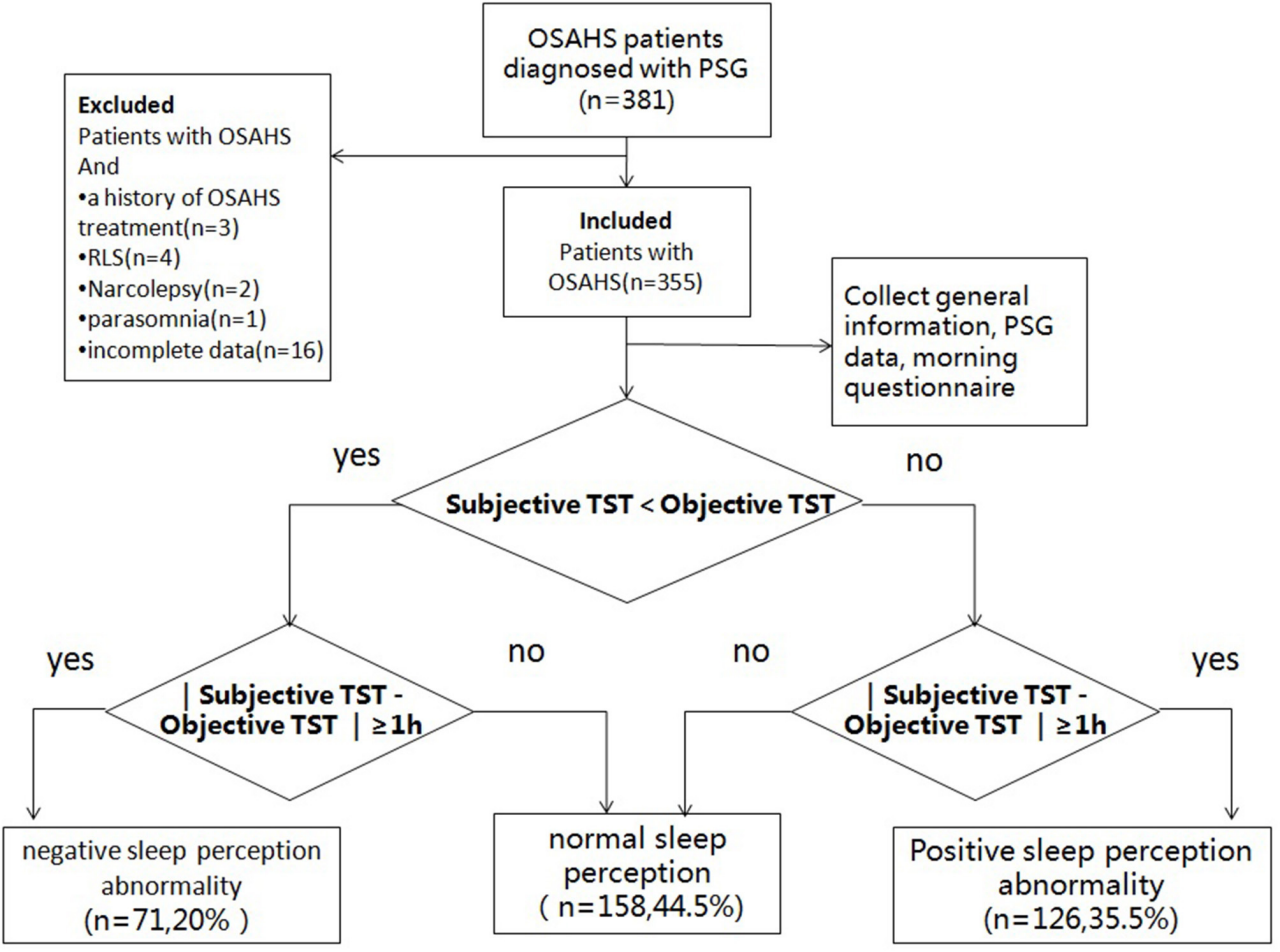
Study enrolment flow. OSAHS, obstructive sleep apnea–hypopnea syndrome; PSG, polysomnography; RLS, restless legs syndrome; TST, total sleep time.

The study subjects were instructed to fill out an application form first, so that the patients' general information (name, sex, age, chief complaint, body mass index, personal history, neck circumference, waist and hip circumference, blood pressure, complication, and so on) were collected before PSG. Patients were also instructed to fill in self-report questionnaires before the PSG for screening patients with neurological conditions and the morning questionnaire after the PSG. The study enrollment is showed in Figure 1. Strict blood pressure measurements were performed before and after the PSG.

This study was approved by the medical ethics committee of the Shengjing Hospital of China Medical University, and written informed consent was obtained from all patients or their next of kin.

#### Self-Report Questionnaires

Depressive symptoms were measured using the Beck self-rating depression questionnaire (6), which is a 21-item self-report questionnaire. Each item is rated on a scale of 0–3. Total scores range from 0 to 63, with a higher score reflecting more depressive mood. The cutoff value reflecting clinical levels of depression is 15. Anxiety symptoms were measured by GAD-7 anxiety screening scale (7), which has seven questions measuring severity of anxiety symptoms. Total scores range from 0 to 21, with a higher score reflecting more anxiety, and those with scores ≥ 5 were excluded.

#### Morning Questionnaire

A questionnaire was filled out after the patient woke up. The patient's subjective perception was used to evaluate his or her sleep progress and sleep quality through 7 questions: (1) How long did it take you to fall asleep last night? (from going to bed to falling asleep); (2) How many times did you wake up last night?; (3) How long were you awake last night?; (4) How long did you sleep last night?; (5) Evaluate your sleep quality last night (A, very good; B, good; C, poor; and D, very poor); (6) How was your sleep last night compared with your previous sleep? (good; similar; poor); and (7) sleep time of about () minutes per night in the last month.

#### Polysomnography Monitoring

All subjects were monitored using a Philips Alice 6 PSG monitor. Items were recorded according to American Academy of Sleep Medicine (AASM) recommendations. All subjects underwent at least 7 h of sleep monitoring at night. On the day of the test, patients were instructed not to drink caffeinated beverages or alcohol, not to use sleep medications, and not to take naps. The results were processed by computer and then manually judged according to AASM version 2.3 (8). Score arousal during sleep stages non-rapid eye movement (NREM) or REM if there is an abrupt shift of electroencephalography (EEG) frequency including alpha, theta, and/or frequencies >16 Hz (but not spindles) that lasts at least 3 s, with at least 10 s of stable sleep preceding the change. Arousal (with respiratory events and oxygen desaturations/with limb movement) was considered to be present when there was a sequence of respiratory events and oxygen desaturations/limb movement lasting at least 10 s to cause arousal from sleep. An arousal that was not caused by respiratory events, oxygen desaturations, or limb movement can be considered a spontaneous arousal. An apnea was defined as > 90% reduction in SpO_2_ for at least 10 s. A hypopnea was defined as a reduction in airflow (>30%) for at least 10 s with oxygen desaturation (≥4%) or arousal. The apnea–hypopnea index (AHI) was calculated as the average number of apneas and hypopneas per hour of sleep. TS90% was the percentage of time spent in sleep below 90% oxygen saturation.

### Diagnostic Criteria

#### Obstructive Sleep Apnea–Hypopnea Syndrome Diagnostic Criteria

According to the *International Classification of Sleep Disorders* (9) published by the AASM in 2014, patients complained of snoring at night, had symptoms of sleepiness during the day, or were reported to regularly snore during sleep by a witness. Their AHI was higher than 5 times/h on the basis of the PSG detection.

#### Sleep Perception

At present, there is no unified diagnostic standard for sleep perception. According to the diagnostic criteria of paradoxical insomnia (10), an absolute value of sleep misperception (subjective TST-objective TST) >60 min can be defined as sleep perception abnormality. We assume that sleep perception discrepancy < −60 min is an underestimation of TST [negative sleep perception abnormality (NSPA)]; sleep perception discrepancy > 60 min is an overestimation of TST [positive sleep perception abnormality (PSPA)]; −60 min ≤ sleep perception discrepancy ≤ 60 min belongs to normal sleep perception (NSP). We define sleep perception as follows: subjective TST/objective TST (4).

### Statistical Analysis

Statistical analysis was performed using SPSS 22.0 statistical software. To test normality assumptions, the Shapiro–Wilk test was used. The measurement data were expressed as mean value ± standard deviation ($\bar{\text{x}}$ ± s). The comparison between the two groups used independent samples *t*-test, and the comparison between multiple groups used one-way ANOVA. Categorical variables were expressed as n (%). Categorical variables were analyzed using chi-square tests or Fisher exact test. Abnormal distribution data were expressed in quartiles. Nonparametric tests were used for comparison between groups; *post hoc* analyses were conducted using Kruskal–Wallis test; correlation analysis was performed using Spearman correlation coefficient analysis. Multiple linear regression analysis was performed to determine the relationships between sleep perception and various PSG indicators; selection of independent variables was conducted using backward stepwise. The difference was statistically significant with *P* < 0.05.

## Results

### Comparison of General Clinical Data Among Obstructive Sleep Apnea–Hypopnea Syndrome Patients With Different Sleep Perception Types

Clinical characteristics of OSAHS patients are summarized in Table 1. Of the 355 OSAHS patients, 158 (44.5%) presented NSP with an average age of 42 years, 71 (20%) presented NSPA (underestimation of TST) with an average age of 47 years, and 126 (35.5%) presented PSPA (overestimation of TST) with an average age of 41 years. There were significantly differences in age among the three groups (*P* < 0.05). Mean age was higher in the NSPA.

**Table 1:** Comparison of general clinical data of the three groups of OSAHS patients with different types of sleep perception.

		**NSPA (*n* = 71, 20%)**	**NSP (*n* = 158, 44.5%)**	**PSPA (*n* = 126, 35.5%)**	***P***
Age (years)		47 ± 12.37	42.69 ± 12.97	41.8 ± 12.66	0.018
Male, *n* (%)		53 (74.6%)	126 (79.7%)	105 (83.3%)	0.430
BMI (kg/m^2^)		27.21 (4.47)	27.71 (5.77)	28.73 (5.3)	0.169
Neck circumference (cm)		40 (6.25)	40 (5)	41 (5)	0.094
Waistline (cm)		98 (15)	99 (14.75)	100.5 (12)	0.112
Hips (cm)		104 (10)	105 (10)	107 (9.75)	0.174
Usual SBP (mmHg)		132.47 ± 16.88	132.26 ± 18.6	135.65 ± 16.90	0.257
Morning SBP (mmHg)		130.12 ± 17.37	131.86 ± 16.79	132.65 ± 18.40	0.63
Usual DBP (mmHg)		88.03 ± 13.75	86.44 ± 12.01	88.83 ± 11.86	0.276
Morning DBP (mmHg)		88.81 ± 14.33	87.67 ± 13.97	89.46 ± 13.94	0.568
Hypertension	Yes	34 (23.4%)	62 (42.8%)	49 (33.8%)	0.401
	No	37 (17.6%)	96 (45.7%)	77 (36.7%)	
Heart disease	Yes	15 (23.8%)	32 (50.8%)	16 (25.4%)	0.179
	No	56 (19.2%)	126 (43.2%)	110 (37.7%)	
Cerebrovascular disease	Yes	7 (29.2%)	9 (37.5%)	8 (33.3%)	0.497
	No	64 (19.3%)	149 (45%)	118 (35.6%)	

*BMI, body mass index; SBP, systolic blood pressure; DBP, diastolic blood pressure; OSAHS, obstructive sleep apnea–hypopnea syndrome; NSPA, negative sleep perception abnormality; NSP, normal sleep perception; PSPA, positive sleep perception abnormality*.

### Comparison of Physiological Indicators (Polysomnography Indicators) of Obstructive Sleep Apnea–Hypopnea Syndrome Patients With Different Types of Sleep Perception

Objective TST and subjective TST of three groups were shown in Table 2. We observed the least objective TST in PSPA group. With regard to PSG indicators, there were significant differences in the number of spontaneous arousals and the oxygen desaturation index (ODI) of OSAHS patients with different types of sleep perception (*P* < 0.05). There was no significant difference in N1, N2, N3, and REM sleep among the three groups; and there were significant differences in REM latency, sleep efficiency, and wake after sleep onset (WASO) (*P* < 0.05). Further *post hoc* test of PSG indicators in the three groups has found that patients with PSPA had fewer spontaneous arousals. PSPA group had significantly lower REM latency and sleep efficiency and more WASO (see Table 2).

**Table 2:** Comparison of PSG indicators in the three groups of OSAHS patients with different types of sleep perception.

	**NSPA (*n* = 71, 20%)**	**NSP (*n* = 158, 44.5%)**	**PSPA (*n* = 126, 35.5%)**	***P***	***post-hoc***
Objective TST (min)	419 (64.5)	394 (119.88)	353.5 (125.13)	<0.001	_
Subjective TST (min)	300 (180)	420 (120)	480 (120)	<0.001	_
Total wake times (events)	9 (13.25)	9 (12)	10 (15)	0.346	_
Arousal times (with respiratory events)	7 (11)	5 (10.25)	3.5 (9.5)	0.069	_
Arousal times (with respiratory events and oxygen desaturations)	37 (133)	42.5 (101.25)	47.5 (112.25)	0.787	_
Arousal times (with limb movement)	27 (57)	30.5 (39.25)	23.5 (42.5)	0.358	_
Spontaneous arousal times (events)	36.5 (47.5)	30 (36)	19 (30)	0.003	PSPA < NSP*
Number of awakenings >15 s (events)	17.5 (26.5)	17 (27)	14 (23.3)	0.77	_
Arousal index (events)	24.6 (33.3)	23.1 (30.1)	25.3 (31.2)	0.885	_
Total arousal times (events)	156 (216)	127 (164)	118 (185.3)	0.386	_
AHI (events/h)	37.6 (43.0)	39.5 (39.9)	47.6 (38.4)	0.123	_
Mean oxygen saturation (%)	95 (3)	95 (2)	95 (4)	0.317	_
ODI (events/h)	32.9 (51.5)	31.4 (49.4)	48.4 (54.2)	0.046	No significant*
LSaO_2_ (%)	81.5 (16)	80 (17)	79 (23)	0.17	_
Severity degree					
mild	16 (30.2%)	23 (43.4%)	14 (26.4%)	0.182	_
moderate	10 (15.6%)	33 (51.6%)	21 (32.8%)		
severe	45 (18.9%)	102 (42.9%)	91 (38.2%)		
Sleep latency (min)	29.3 (23.5)	26.65 (22.9)	27.95 (24.28)	0.307	_
N2 latency (min)	11 (15)	8.75 (12.5)	8.5 (10.75)	0.37	_
N3 latency (min)	68 (193)	52.5 (141.25)	37 (136.75)	0.211	_
REM latency (min)	140.5 (150)	165.5 (132.5)	131.5 (170)	0.027	PSPA < NSP*
REM (%TST)	8.9 (9.6)	7.5 (6.85)	7.9 (9.6)	0.81	_
N1 (%TST)	37 (19)	32.2 (18.73)	32.1 (17.7)	0.682	_
N2 (%TST)	51.4 (12.2)	54.85 (13.78)	53.9 (14)	0.231	_
N3 (%TST)	2.7 (6.2)	2.65 (5.3)	2.85 (5.6)	0.997	_
TS90% (%)	3.62 (21.48)	2.9 (18.51)	8.16 (37.22)	0.065	_
PLMI (events/h)	6.7 (11.6)	6.2 (11.23)	9.6 (12.7)	0.421	_
Sleep efficiency (%)	78 (18)	78 (20)	71 (21)	<0.001	PSPA < NSP, NSPA*
WASO (min)	89.6 (115.7)	87 (92.8)	123.35 (116.9)	0.003	PSPA > NSP*

*TST, total sleep time; AHI, apnea–hypopnea index; ODI, oxygen desaturation index; LSaO_2_, lowest oxygen saturation; REM (%TST), the percentage of REM sleep to TST; N1 (%TST), N2 (%TST), and N3 (%TST), the percentage of N1 period, N2 period, and N3 period to TST; TS90%, the percentage of time when oxygen saturation is lower than 90% to TST; PLMI, periodic limb movement index; WASO, weak after sleep onset; PSG, polysomnography; OSAHS, obstructive sleep apnea–hypopnea syndrome; NSPA, negative sleep perception abnormality; NSP, normal sleep perception; PSPA, positive sleep perception abnormality*.

^*^In *post hoc* test of PSG indicators in the three groups, there were significant differences between the PSPA and NSP groups in spontaneous arousal times (*P* = 0.003), REM latency (*P* = 0.027), sleep efficiency (*P* < 0.001), and WASO (*P* = 0.002), but no significant difference in oxygen desaturation index (*P* = 0.06). There was a significant difference between the PSPA and NSPA groups in sleep efficiency (*P* = 0.004). There was no significant difference between the NSPA and NSP groups in any PSG indicator (*P* > 0.05).

### Correlation Analysis Between Sleep Perception and Sleep Parameters in Patients With Obstructive Sleep Apnea–Hypopnea Syndrome

Table 3 reveals that sleep perception was positively correlated to AHI, ODI, TS90%, and WASO and negatively correlated to arousal times with respiratory events, spontaneous arousal times, and sleep efficiency. Multivariate linear regression was conducted using the above variables. Selection of independent variables was conducted using backward stepwise. As shown in Table 4, two separate linear regression analyses after adjusting for age and sex revealed that sleep perception was related to LSaO_2_, TS90%, sleep efficiency, and WASO.

**Table 3:** Correlation analysis between sleep perception and PSG indicators.

	**Sleep perception (subjective TST/objective TST)**	
	***r***	***P***
Total wake times (events)	0.098	0.064
Arousal times (with respiratory events)	−0.153	0.004
Arousal times (with respiratory events and oxygen desaturations)	0.029	0.585
Arousal times (with limb movement)	−0.074	0.163
Spontaneous arousal times (events)	−0.186	<0.001
Arousal index (events)	0.024	0.647
Total arousal times (events)	−0.085	0.111
AHI (events/h)	0.121	0.022
Mean oxygen saturation (%)	−0.103	0.052
ODI (events/h)	0.139	0.009
LSaO_2_ (%)	−0.11	0.038
Sleep latency (min)	−0.059	0.264
N2 latency (min)	−0.055	0.303
N3 latency (min)	−0.073	0.169
REM latency (min)	−0.095	0.074
REM (%TST)	−0.01	0.857
N1 (%TST)	−0.011	0.836
N2 (%TST)	0.008	0.874
N3 (%TST)	0.018	0.737
TS90% (%)	0.149	0.005
PLMI (events/h)	0.04	0.465
Sleep efficiency (%)	−0.221	<0.001
WASO (min)	0.179	0.001

*PSG, polysomnography; TST, total sleep time; AHI, apnea–hypopnea index; ODI, oxygen desaturation index; LSaO_2_, lowest oxygen saturation; REM, rapid eye movement; PLMI, periodic limb movement index; WASO, wake after sleep onset*.

**Table 4:** Multiple linear regression analysis of sleep perception and PSG indicators.

	**Variables**	**Beta**	***P***
Regression 1	Age (years)	−0.193	<0.001
	Sex, *n* (%)	−0.048	0.351
	LSaO_2_ (%)	−0.107	0.035
	Sleep efficiency (%)	−0.800	<0.001
	WASO (min)	−0.437	<0.001
Regression 2	Age (years)	−0.159	0.001
	Sex, *n* (%)	−0.004	0.930
	TS90% (%)	0.301	<0.001
	Sleep efficiency (%)	−0.803	<0.001
	WASO (min)	−0.477	<0.001

*PSG, polysomnography; LSaO_2_, lowest oxygen saturation; WASO, wake after sleep onset*.

## Discussion

Sleep perception is a state feature, including both time and quality, which is an individual's evaluation of the sleep state. The abnormal sleep perception is mainly manifested in the differences in subjective and objective sleep time. Time perception belongs to the category of psychology. With regard to time research in psychology, there are generally three types of understanding of the difference between physical time and psychological time: biological models, cognitive models, and comprehensive models. Regardless of whether it is from a biological perspective or cognitive perspective, time perception is a mixture of internal and external factors (11). According to the time perception models, the sleep perception judgment of people with sleep disorders may be affected by many factors. Some studies have suggested that the causes of the difference between subjective perception and objective perception include individual personality characteristics, emotions, and sleep physiology, such as the individual's metabolism during sleep, the effects of EEG during sleep (increased α-δ waves), cyclic alternation patterns, and sleep staging conditions (12).

Some scholars once compared the sleep perception of normal people, patients with OSAHS, and people with insomnia, and they believed that normal people's sleep perception was the best, insomnia patients had the worst sleep perception, and OSAHS patients' sleep perception was in the middle (3). Our study grouped OSAHS patients according to the types of sleep perception abnormalities and found that 55.5% of OSAHS patients had sleep perception abnormalities, including both negative sleep perception abnormalities (20%) and positive sleep perception abnormalities (35.5%). We found that there was a negative correlation between sleep perception and the number of spontaneous arousal, which means that the better the sleep perception, the fewer the number of spontaneous arousals. Hermans et al. (13) found that the misperception of SL was related to the increase in N1 sleep period and the increase in WASO, which indicates that light sleep and fragmentation of sleep are associated with abnormal sleep perception in individuals. However, our study also found that sleep perception had no correlation with the patient's arousal index. This result suggests that sleep perception abnormalities in OSAHS patients may only be related to the individual's spontaneous arousal, but not related to arousal caused by other factors in sleep (respiratory events, oxygen desaturation, and limb movement events). Some studies (12) also believed that when respiratory events happen in OSAHS patients, the reduction of blood oxygen saturation and arousal will not interfere with sleep perception. Austin Saline (14) and others believed that when OSAHS patients filled out the morning questionnaire, they generally estimated the subjective TST on the basis of the subjective SL, and their estimates of the SL might not be accurate, so Austin Saline adjusted the TST for analysis, finding that sleep fragmentation could not explain abnormal sleep perception in the SL and TST. More evidence is needed to determine whether respiratory events in OSAHS patients can affect their sleep perception.

As early as 1992, researchers have studied the abnormal sleep perception of insomnia patients (15), and they found that most of the patients with insomnia underestimate their sleep time. The researchers believed that abnormal sleep perception is a precursor of objective insomnia and that patients with negative sleep perception may be predicted to have concomitant insomnia. It is an established finding that insomnia patients have difficulty recognizing sleep state. However, we found that the NSPA group has higher sleep efficiency, which elucidate that OSAHS patients with negative sleep perception were not associated with insomnia. In our study, we compared the respiratory-related indicators detected by PSG of OSAHS patients with three types of sleep perception, and we found that there is no significant difference in respiratory-related indicators. OSAHS patients with PSPA had a higher oxygen desaturation index (ODI), but no significant difference in *post hoc* test. A study by Jelina Castillo et al. (16) compared sleep perception after polysomnographic monitoring with sleep perception after pressure titration, and they found that fewer OSAHS patients overestimate TST after pressure titration therapy, which indicates that after the reduction in respiratory events, the sleep perception of patients who originally overestimated TST was improved. However, their study did not find any correlation between sleep monitoring indicators and sleep perception abnormalities, which led us to consider whether the positive and negative directions of patients' sleep perception will hint respiratory events. In our results, sleep perception was negative related to LSaO_2_ and positive related to TS90%. Patients with positive sleep perception are likely to suffer more severe hypoxia and need early treatment.

Sleep structure is another important indicator in monitoring sleep. Normal adults have a periodic sleep the entire night. The sleep cycle starts with NREM sleep and ends with REM sleep. There are about four to six sleep cycles per night. We compared the sleep stages and the SL of patients with three types of sleep perception. It was found that there was no difference in the proportion of sleep in each period of sleep. REM latency showed significant discrepancy. The OSAHS patients with abnormal sleep perception had shorter REM latency. As they entered the REM period faster and the individual sleep cycle was relatively shorter, there were more periodic sleep cycles during overnight sleep. Hyunwoo Nam et al. (17) believed that the delay of the sleep cycle plays an important role in patients with abnormal sleep perception, which is contradictory to our conclusions. We assume that the transitions of sleep cycle affect sleep perception, and the judgment of sleep cycles is accomplished through EEG activity. Previous studies have found that there is a connection between sleep perception and EEG, which reflects that the amplitude of low-frequency waves in the prefrontal cortex of negative sleep perception patients is higher than that of the normal control group, and the activity of alpha wave and gamma wave is strengthened (18). In 1982, MG Terzano et al. proposed an alternating pattern of EEG circulation during sleep, which is a periodic EEG change in NREM sleep. It is negatively correlated with subjective perceived sleep quality (19, 20). These results support the hypothesis that differences in sleep perception relate to the increase in periodic EEG activity. In addition, our result found there was a significant increase of Stage W after sleep onset (WASO) in PSPA. In the sleep cycle, humans have transient wakefulness (Stage W), which is undetectable. Hermans hypothesized that a sleep fragment needs to have a certain duration to be perceived as sleep. Sleep onset under 10 min is perceived as wake (12). Conversely, a short wakefulness during sleep state might be perceived as sleep. Therefore, future work could focus on the relationship between sleep–wake stage transition and sleep perception.

The limitation of our study is that our sleep parameters lack in-depth analysis of EEG data. EEG parameters like spindle wave might play a role in sleep perception. Some researchers suggested sleep spindles might inhibit sensory processing, especially the disengagement of disrupting and intrusive stimuli. Reduced density in sleep spindles might in turn enhance underestimation of sleep (21). EEG of OSAHS patients and their role toward the altered sleep perception is our future research direction.

Sleep perception abnormalities in OSAHS patients are very common. This study found that sleep perception in OSAHS patients is correlated with spontaneous arousal and WASO. Patients with positive sleep perception abnormalities had more severe nighttime hypoxia. Whether the respiratory event affects sleep perception and its mechanisms still need to be supported by more research evidence. Our results open up avenues to further study the sleep perception of OSAHS patients. Noteworthy questions include the following: Does reducing respiratory events in OSAHS patients affect their sleep perception? Do patients with negative sleep perception abnormalities have concomitant insomnia? What role does EEG play in OSAHS? Clinical analysis should pay attention to different sleep perception subtypes of OSAHS patients. Sometimes, the subjective evaluation of symptoms of patients will mask the actual condition. Therefore, timely personalized treatment, including sleep health education and cognitive behavior, is needed.

## Data Availability Statement

All datasets generated for this study are included in the article/Supplementary Material.

## Ethics Statement

The studies involving human participants were reviewed and approved by Ethics Committee of Shengjing Hospital of China Medical University. The patients/participants provided their written informed consent to participate in this study.

## Author Contributions

YL: substantial contributions to the conception and design of the work and wrote this paper. HT, YY, and YZ: they did some work about the acquisition, analysis or interpretation of data. LX: provided us a favorable research environment.

## Conflict of Interest

The authors declare that the research was conducted in the absence of any commercial or financial relationships that could be construed as a potential conflict of interest.
